# NURSING FACILITY RESPONSES TO GROWING CONSUMER COMPLAINTS

**DOI:** 10.1093/geroni/igae098.2800

**Published:** 2024-12-31

**Authors:** Robert Applebaum, John Bowblis, Matt Nelson, Oksana Dikhtyar

**Affiliations:** Miami University, Oxford, Ohio, United States; Miami University, Oxford, Ohio, United States; Miami University, Oxford, Ohio, United States; Miami University, Oxford, Ohio, United States

## Abstract

Nursing home consumers can file a complaint to regulators regarding such areas as quality of care or resident rights. The number of formal complaints filed has increased at dramatically. Between 2016 and 2021, Ohio saw a 40% increase in the number of complaints. A number of factors are associated with complaints including staffing levels and staff and leadership turnover. The increase in complaints is an indicator that residents and families do not feel satisfied with their nursing home experience, and it is also a huge strain on the nursing home inspection process. Last year in Ohio fewer than 40% of required nursing home recertification surveys were completed in the required window. The critical question then becomes, are there things that can be done by facilities to reduce the number of complaints filed with regulators. Using data from the 2023 Biennial Survey of Long-Term Care Facilities completed in Ohio on all licensed nursing homes, this paper examines the strategies used by nursing homes to respond to the growing number of complaints. Approaches examined include such areas as having a dedicated staff member to examine complaints or building a review of complaints into the facility quality improvement process. The survey also presents findings about the interactions with the Department of Health survey teams. Data suggest there is a long lag between a complaint visit and an adjudication of the complaint. The paper concludes with recommendations for facilities and states as they respond to the growing number of complaints experienced across the nation.

